# Stress-specific 14-3-3–client modules in digestive cancers: an evidence-graded review of adaptive survival and therapy resistance

**DOI:** 10.1080/15384047.2026.2710413

**Published:** 2026-07-27

**Authors:** Rudong Li, Zhipeng Zhao, Xudong Wang

**Affiliations:** a Department of Gastrointestinal Nutrition and Hernia Surgery, The Second Hospital of Jilin University, Changchun, Jilin, People's Republic of China

**Keywords:** 14-3-3 proteins, digestive cancer, therapy resistance, stress granules, unfolded protein response, autophagy, anoikis resistance, YWHAZ

## Abstract

14-3-3 proteins are phosphoserine- and phosphothreonine-binding adaptors that regulate client localization, stability and activity under cellular stress. This narrative review synthesizes stress-specific 14-3-3–client modules in gastric, colorectal, pancreatic, hepatocellular and biliary cancers. Modules were classified as high, moderate, early/context-dependent or background according to mechanistic evidence, functional perturbation, client mapping, treatment-state validation and clinical association. In gastric cancer, G3BP stress granule assembly factor 1 (G3BP1) cooperates with YWHAZ-encoded 14-3-3ζ to retain pro-apoptotic Bax in the cytoplasm. In colorectal cancer, SFN-encoded 14-3-3σ restricts Yin Yang 1 (YY1), sustaining the unfolded protein response and chemotherapy tolerance. In pancreatic cancer, SFN-encoded 14-3-3σ interacts with Yes-associated protein 1 (YAP1) to promote ribonucleotide reductase expression and gemcitabine resistance. In hepatobiliary cancers, SFN-related modules support anoikis resistance but remain context dependent. Clinically relevant units are stress-specific 14-3-3–client complexes rather than total 14-3-3 expression.

## 1. Introduction and scope

Digestive cancers remain major contributors to cancer morbidity and mortality worldwide.^1^ Disease-specific guidelines for gastric cancer (GC) and colorectal cancer (CRC) illustrate how modern multimodality care has improved selected outcomes while leaving adaptive resistance and residual disease as major limitations.^2^ ^,^ ^3^ Reviews and guidelines for pancreatic ductal adenocarcinoma (PDAC), hepatocellular carcinoma (HCC) and cholangiocarcinoma (CCA) show a similar pattern in which systemic therapy advances coexist with profound stress-adaptive resistance.^4-7^ Tumor cells in GC, CRC, PDAC, HCC and CCA are repeatedly exposed to drug pressure, hypoxia, nutrient limitation, stromal interaction, immune selection and detachment from extracellular matrix. These pressures converge on stress-response programs that determine whether cells undergo apoptosis, enter dormancy, remodel metabolism, acquire invasive plasticity or persist as therapy-resistant disease.^8-10^

This review uses the term digestive cancers pragmatically rather than as a claim that all gastrointestinal and hepatobiliary tumors share one uniform biology. The scope is deliberately restricted to GC, CRC, PDAC, HCC and CCA because these cancer types currently provide the clearest direct evidence for 14-3-3-centered stress-adaptation modules. Evidence from esophageal cancer, gallbladder cancer and other digestive sites is considered only when it informs a defined 14-3-3 stress mechanism. This boundary is important because 14-3-3 proteins are ubiquitous adapters, and observations from one cancer type cannot be transferred to another without mechanistic support.

14-3-3 proteins are acidic dimeric phosphoserine/phosphothreonine-binding adapters that regulate client-protein localization, stability, activity and complex formation.^11-15^ In cancer, their biological output is highly context-dependent. A given isoform may support survival under one stress condition yet restrain tumor progression in another cellular state. The central premise of this review is therefore not that 14-3-3 proteins are uniformly oncogenic or tumor suppressive, but that defined 14-3-3–client complexes can convert phosphorylation events into stress-state-specific survival programs.

A module-based narrative evidence strategy was used. Highest priority was assigned to digestive-cancer studies that directly examined a 14-3-3 isoform, a client interaction or a client-localization event in relation to therapy resistance, stress survival, apoptosis, unfolded protein response (UPR), autophagy, anoikis or metastatic adaptation. A second tier comprised digestive-cancer stress-biology studies that did not directly focus on 14-3-3 proteins but explained the relevant stress context. A third tier comprised general 14-3-3 structural or signaling studies^11-13^ ^,^ ^16^ and apoptotic-client studies used to support mechanistic interpretation.^17-20^ This is a narrative review rather than a systematic review; conclusions are therefore evidence-graded and intended as an organizing framework for future validation. Unlike prior reviews that discuss 14-3-3 proteins broadly as oncogenic or tumor-suppressive factors, this review organizes digestive-cancer evidence by stress state, client interaction and the extent of mechanistic, functional and clinical support relevant to biomarker development or therapeutic testing. The aim of this review is to evaluate stress-specific 14-3-3–client modules across digestive cancers, grade the maturity of the supporting evidence, and identify the contexts in which these modules may inform biomarker development or interaction-directed therapy. Accordingly, we focus on defined isoform–client complexes operating under specific stress conditions rather than on total 14-3-3 expression alone.

## 2. Molecular logic of 14-3-3-centered stress adaptation

The human 14-3-3 family includes YWHAB/14-3-3β, YWHAE/14-3-3ε, YWHAG/14-3-3γ, YWHAH/14-3-3η, YWHAQ/14-3-3θ/τ, YWHAZ/14-3-3ζ and SFN/14-3-3σ.^11^ ^,^ ^21^ ^,^ ^22^ These isoforms share a conserved amphipathic groove that recognizes phosphorylated motifs,^14^ ^,^ ^23^ but they differ in expression patterns, dimerization preferences and client networks. This biochemical architecture allows 14-3-3 proteins to operate as localization and complex-assembly switches rather than as linear enzymes.

In stress-exposed cancer cells, this switch-like behavior is therapeutically relevant. Chemotherapy can induce DNA damage signaling, stress granules (SGs), UPR, apoptotic priming and mitochondrial stress. Detachment from extracellular matrix activates receptor trafficking, extracellular signal-regulated kinase (ERK) signaling, oxidative stress and mitochondrial apoptosis. Hypoxia and nutrient deprivation increase UPR and autophagy pressure. In each setting, 14-3-3 binding may determine whether a phosphorylated client enters the nucleus, remains in the cytoplasm, is degraded, reaches mitochondria or participates in a stress-associated condensate.^12-14^ ^,^ ^16^

Client localization is a recurring theme. Phosphorylated B-cell lymphoma 2 (BCL-2)-associated agonist of cell death (BAD) binds 14-3-3 proteins, preventing BAD from antagonizing anti-apoptotic BCL-2 family members.^17^ ^,^ ^24^ 14-3-3 proteins can also interact directly with BCL-2-associated X protein (Bax) and negatively regulate Bax activity.^18^ In CRC, SFN can regulate the nucleocytoplasmic distribution of Yin Yang 1 (YY1) and transcription factor EB (TFEB), a key regulator of lysosomal biogenesis and autophagy, with distinct effects on the UPR and autophagic activity.^25^ ^,^ ^26^ In HCC, SFN can inhibit epidermal growth factor receptor (EGFR) degradation and sustain ERK1/2 signaling during detachment stress.^27^ Thus, the relevant therapeutic unit is rarely total 14-3-3 abundance; it is the active 14-3-3–client complex in a defined stress state.

In this framework, human clinical association refers to evidence from patient-derived specimens or clinically annotated datasets linking module-related expression, localization, complex activity or stress-output markers to treatment response, recurrence, metastatic progression or survival. Such evidence is considered supportive but does not, on its own, establish causality or clinical utility. Modules were classified as high when direct digestive-cancer mechanistic evidence, functional perturbation, stress-state validation and human clinical association were all available; moderate when direct mechanistic and functional evidence existed but clinical validation remained limited; early/context-dependent when digestive-cancer-specific associative or functional evidence existed but client mapping, treatment-state validation or clinical correlation was incomplete; and background when the proposed mechanism was derived mainly from general 14-3-3 biology. Translational maturity therefore reflects how far a proposed module has progressed from association to a stress-matched, causally supported mechanism with potential relevance to biomarker stratification or interaction-directed therapeutic testing; it does not indicate biological importance or demonstrate clinical efficacy.

## 3. Evidence-graded cancer-type modules

**Figure 1.**
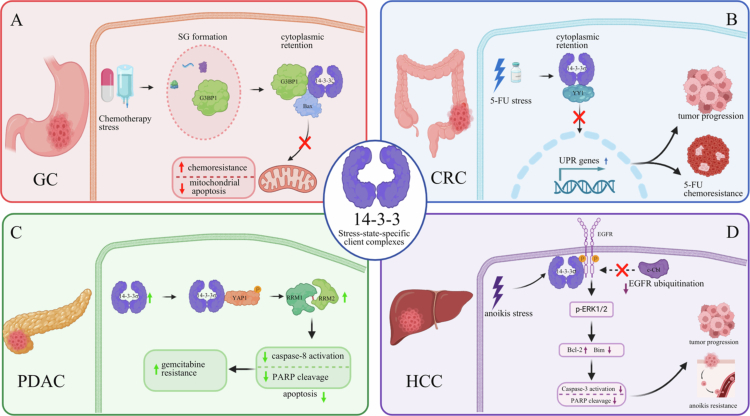
Core 14-3-3–client modules in digestive cancers. (A) GC: G3BP1–YWHAZ retains Bax in the cytoplasm and limits chemotherapy-induced apoptosis. (B) CRC: SFN–YY1 sustains the unfolded protein response and chemotherapy tolerance. (C) PDAC: SFN–YAP1 promotes RRM1/RRM2 expression and gemcitabine resistance. (D) HCC: SFN stabilizes EGFR–ERK1/2 signaling and promotes anoikis resistance.

The core 14-3-3-centered stress-response modules discussed in this section are summarized in Figure 1.

### 3.1. GC: SG-associated G3BP1–YWHAZ–Bax

SGs are non-membranous cytoplasmic ribonucleoprotein assemblies that form during oxidative stress, nutrient deprivation, heat shock and chemotherapy exposure.^28-30^ In cancer, SGs can transiently repress translation, preserve stress-adaptive transcripts, reorganize signaling proteins and buffer apoptosis.^31^ ^,^ ^32^ Among digestive cancers, GC provides one of the clearest examples in which SG biology, 14-3-3 adapter binding and mitochondrial apoptosis are mechanistically connected.

Zhao et al. reported that G3BP stress granule assembly factor 1 (G3BP1), a core SG nucleator, interacts with YWHAZ/14-3-3ζ in GC cells.^33^ G3BP1 depletion increased chemotherapy sensitivity and enhanced apoptosis-associated molecular changes. Mechanistically, the G3BP1–YWHAZ interaction promoted cytoplasmic retention of Bax, limiting Bax-mediated mitochondrial apoptosis. Clinically, tumors with high G3BP1 and high YWHAZ were associated with the poorest outcomes after adjuvant chemotherapy, suggesting that this module may mark limited benefit from chemotherapy.^33^ This combination of physical interaction, cell-fate effect, Bax localization and clinical association makes G3BP1–YWHAZ–Bax the highest-maturity module in the review.

The GC module is best interpreted as a client-sequestration mechanism rather than simply as increased YWHAZ expression. In this module, G3BP1 provides an SG-associated scaffold, YWHAZ/14-3-3ζ supplies the adapter function, and Bax acts as the pro-apoptotic client whose mitochondrial access determines apoptotic output. Thus, SG disruption, G3BP1–YWHAZ interaction assays and Bax localization measurements are more informative than measuring YWHAZ abundance alone.

Microenvironmental evidence is supportive but less mature. A study of Banxia Xiexin Decoction reported that exosomes derived from bone marrow mesenchymal stromal cells (BMSCs) enhanced oxaliplatin resistance in GC, promoted SG formation and strengthened the G3BP1–YWHAZ interaction.^34^ Because exosomes are a subtype of extracellular vesicles (EVs), this study is most appropriately interpreted as supportive evidence for EV-mediated amplification rather than as co-primary evidence for the core GC mechanism. Independently, TMEM65 has been reported to interact with and stabilize YWHAZ, thereby activating phosphoinositide 3-kinase (PI3K)–AKT-mechanistic target of rapamycin (mTOR) signaling and promoting GC tumorigenesis.^35^ The TMEM65–YWHAZ pathway is mechanistically distinct from SG-associated Bax sequestration but supports the broader concept that YWHAZ can function as a survival-associated adapter node in GC.

### 3.2. CRC: SFN–YY1–UPR and context-dependent SFN biology

The UPR is activated when the protein-folding capacity of the endoplasmic reticulum (ER) is overwhelmed. Its protein kinase R-like endoplasmic reticulum kinase, inositol-requiring enzyme 1α and activating transcription factor 6 arms regulate translation, chaperone expression, ER-associated degradation, redox adaptation, autophagy and apoptosis.^36-38^ In tumors, moderate or chronic UPR activation can support survival under nutrient deprivation, hypoxia and chemotherapy, whereas severe or unresolved ER stress can trigger cell death.^39^ ^,^ ^40^ CRC therefore provides a biologically plausible context in which proteotoxic stress and chemotherapy tolerance may converge.^41^

The most direct CRC 14-3-3-UPR mechanism is the SFN–YY1 axis. Lonare et al. showed that SFN/14-3-3σ binds YY1 and restricts YY1 to the cytoplasm.^25^ Because nuclear YY1 represses UPR-related genes, SFN-mediated cytoplasmic retention maintains UPR gene expression. Loss of SFN reduced tumor formation and increased chemotherapy sensitivity, while genetic or pharmacological interference with UPR signaling sensitized colon cancer cells to chemotherapy.^25^ This module has high mechanistic maturity but moderate overall evidence maturity because treatment-annotated clinical validation is lacking.

SFN is not uniformly oncogenic in CRC. It is a p53-regulated G2/M checkpoint effector, is required to prevent mitotic catastrophe after DNA damage and can function as an intestinal tumor suppressor.^42-45^ More recent CRC studies further reinforce this context dependence. Transforming growth factor beta (TGF-*β*)/SMAD family member 4 (SMAD4) signaling can induce SFN, which sequesters TFEB in the cytoplasm, thereby restraining autophagy and supporting epithelial features in CRC.^26^ Separately, the SFN–NEDD4-like E3 ubiquitin protein ligase (NEDD4L) axis promotes ubiquitination and degradation of hypoxia-inducible factor 1 alpha (HIF-1α), suppressing hypoxia-induced metastasis and angiogenesis in CRC.^46^ These observations explain why global SFN inhibition may be unsafe or biologically misleading.

YWHAH/14-3-3η introduces another CRC autophagy-related module. A recent study reported that YWHAH regulates autophagy through mitogen-activated protein kinase (MAPK)/ERK signaling and influences CRC migration and invasion.^47^ This finding supports inclusion of YWHAH in the broader 14-3-3-autophagy discussion, but its evidence maturity is lower than that of the SFN–YY1–UPR module because clinical validation and therapy-resistance relevance remain less developed. YWHAH–MAPK/ERK-autophagy is therefore treated as an early CRC module rather than as a pan-digestive therapeutic target. Thus, SFN should be interpreted as a state-dependent adapter whose therapeutic relevance depends on the dominant client complex, rather than as a uniform target in CRC.

### 3.3. Pancreatic cancer: SFN–YAP1–RRM1/RRM2 and gemcitabine tolerance

PDAC is characterized by dense stroma, metabolic stress, poor drug delivery and profound therapy resistance.^4^ ^,^ ^5^ Gemcitabine remains a foundational nucleoside analog in PDAC treatment, but resistance frequently emerges through drug transport, nucleotide metabolism, DNA damage tolerance, apoptotic threshold and stromal mechanisms.^48-50^ Because gemcitabine activity depends on DNA incorporation and nucleotide pools, ribonucleotide reductase catalytic subunit M1 (RRM1) and ribonucleotide reductase regulatory subunit M2 (RRM2) are important determinants of gemcitabine response.^51-53^

SFN/14-3-3σ has been linked to poor prognosis and resistance to radiation and chemotherapy in PDAC.^54^ Qin et al. subsequently showed that 14-3-3σ is upregulated in PDAC cells with acquired gemcitabine resistance. Mechanistically, 14-3-3σ regulates and interacts with Yes-associated protein 1 (YAP1), suppresses gemcitabine-induced caspase-8 activation and apoptosis, and promotes RRM1/RRM2 expression.^55^ This defines a moderate-maturity PDAC stress-survival module: direct mechanistic evidence is available, but validation in patient-derived organoids, stromal co-culture systems and clinically annotated resistant tumors remains necessary.

YAP1 is a transcriptional coactivator and major downstream effector of the Hippo pathway, with broad roles in mechanotransduction, cellular plasticity, metabolic adaptation and therapy resistance.^56-58^ In PDAC, YAP1-associated signaling has also been linked to gemcitabine resistance.^59^ The SFN–YAP1–RRM1/RRM2 module is therefore biologically coherent because it connects a 14-3-3 adapter, a stress-adaptive transcriptional co-regulator and enzymes that modulate nucleotide pools during gemcitabine exposure. However, PDAC resistance is not reducible to this module alone. Therapeutic interpretation needs to consider drug delivery, tumor–stroma interaction, KRAS-driven metabolic rewiring, apoptotic priming and alternative DNA damage response mechanisms.

The translational path is strongest when focused on module-enriched models rather than unselected PDAC lines. Candidate biomarkers include SFN expression, YAP1 localization or activity, RRM1/RRM2 expression, caspase-8 activation and gemcitabine response. SFN–YAP1–RRM1/RRM2-high models may be prioritized for preclinical testing of combinations that target replication-stress tolerance, ribonucleotide reductase, YAP/transcriptional coactivator with PDZ-binding motif (TAZ) activity or apoptosis priming, but these remain preclinical hypotheses.

### 3.4. Hepatobiliary cancers: SFN–EGFR/ERK anoikis-resistance modules and context-dependent evidence

Anoikis is apoptosis induced by loss of extracellular-matrix attachment. Its evasion is necessary for epithelial tumor cells to survive invasion, circulation, peritoneal spread and metastatic colonization.^60-62^ Hepatobiliary cancers encounter detachment stress during vascular invasion, biliary dissemination, stromal invasion and metastatic spread, making anoikis resistance a clinically relevant stress phenotype.

In HCC, the SFN–EGFR/ERK module is the most mechanistically defined hepatobiliary example. Song et al. reported that SFN promotes anoikis resistance by inhibiting EGFR degradation and activating EGFR-dependent ERK1/2 signaling.^27^ This mechanism is notable because SFN supports detachment survival through receptor stability rather than only through direct regulation of apoptotic proteins. The HCC module therefore has moderate maturity: it has direct mechanistic and functional support, but broader patient stratification and therapeutic validation are still needed.

CCA data require more caution. Proteomic analysis of anoikis-resistant CCA cells identified upregulation of 14-3-3σ, and SFN knockdown increased cell death under suspension conditions.^63^ Experimental knockdown of SFN/14-3-3σ suppressed intrahepatic CCA cell migration, invasion and anoikis resistance.^64^ However, earlier clinical work found that 14-3-3σ negatively regulates the cell cycle and that its downregulation is associated with poor outcome in intrahepatic CCA.^65^ These findings do not support a single directional model. Instead, they indicate that SFN may have context-dependent roles in biliary cancers, and that client mapping, phosphorylation status and stress state are required before therapeutic claims can be made.

For hepatobiliary translation, standard adherent two-dimensional proliferation assays are insufficient. Suspension culture, spheroids, organoids, extracellular-matrix remodeling systems, invasion-front sampling and metastatic models are needed to test whether SFN–EGFR/ERK or SFN-associated suspension-survival modules are active. EGFR/ERK inhibition is most plausible where SFN-dependent receptor stabilization and detachment signaling are demonstrated, not as a generic consequence of SFN expression.

The evidence maturity of these modules is summarized in Table 1.

**Table 1.** Evidence-graded 14-3-3-centered stress modules in digestive cancers.

Cancer context	Module	Stress output	Evidence maturity	Key evidence and interpretation
GC	G3BP1–YWHAZ–Bax	SG-associated Bax sequestration; chemoresistance	High	Direct interaction, Bax localization, chemotherapy sensitivity and clinical association support a mature module.^33^ EV/BMSC data are supportive.^34^
GC	TMEM65–YWHAZ	YWHAZ stabilization; PI3K–AKT–mTOR signaling	Moderate	Supports broader YWHAZ survival biology in GC, but is distinct from the SG-Bax mechanism.^35^
CRC	SFN–YY1–UPR	YY1 cytoplasmic retention; UPR maintenance; chemotherapy tolerance	Moderate	Direct SFN–YY1 localization and UPR functional data are strong, but prospective biomarker thresholds remain undeveloped.^25^
CRC	SFN–TFEB	TFEB sequestration; autophagy restraint; epithelial-state regulation	Moderate	Shows context-dependent SFN function and cautions against global SFN inhibition.^26^
CRC	SFN–NEDD4L–HIF-1α	HIF-1α degradation; suppression of hypoxia-associated progression	Moderate	Direct CRC mechanism supports tumor-suppressive SFN biology in hypoxic contexts.^46^
CRC	YWHAH–MAPK/ERK-autophagy	Autophagy-linked migration and invasion	Early/context-dependent	Direct CRC evidence exists, but therapy-resistance relevance and clinical validation are less mature.^47^
PDAC	SFN–YAP1– RRM1/RRM2	RRM1/RRM2 induction; apoptosis suppression; gemcitabine tolerance	Moderate	Direct acquired-resistance model evidence; organoid and patient validation are needed.^54^ ^,^ ^55^
HCC	SFN–EGFR/ERK	EGFR stabilization; ERK activation; anoikis resistance	Moderate	Mechanistically defined HCC detachment-survival module.^27^
CCA	SFN-associated anoikis/invasion	Suspension survival and invasion	Early/context-dependent	Proteomic and functional data exist, but clinical and mechanistic directions are context-dependent.^63-65^
General apoptosis biology	BAD/Bax/BCL-2-family regulation	Apoptotic threshold control	Background	14-3-3–BAD/Bax and BCL-2-family biology support the concept; digestive-cancer-specific claims require direct evidence.^17^ ^,^ ^18^ ^,^ ^66^ ^,^ ^67^

## 4. Cross-module convergence and evidence boundaries

SG formation, the UPR, autophagy, and anoikis resistance are often studied separately, although these stress-response processes frequently coexist in digestive cancer cells. A chemotherapy-exposed GC cell may form SGs, activate ER stress, alter mitochondrial priming and receive exosomal cues from stromal cells. A detached HCC cell may activate EGFR/ERK signaling, modulate mitochondrial apoptosis, alter autophagy and remodel receptor trafficking. A gemcitabine-resistant PDAC cell may tolerate replication stress while engaging YAP1, RRM1/RRM2 and apoptosis escape. 14-3-3 proteins provide a common biochemical logic for these diverse outputs: phosphorylation-dependent client binding changes subcellular access and complex formation.^12^ ^,^ ^14^ ^,^ ^16^

The first convergence point is apoptotic threshold control. SGs can protect cells from acute drug-induced apoptosis, UPR can delay death while proteostasis is restored, autophagy can remove damaged organelles, and anoikis resistance prevents detachment-induced mitochondrial collapse. BCL-2 family proteins, caspases and mitochondrial outer membrane permeabilization provide the core apoptotic machinery for these fate decisions.^19^ ^,^ ^20^ ^,^ ^66^ 14-3-3-dependent regulation of BAD and Bax provides the most relevant 14-3-3 apoptotic-client background.^17^ ^,^ ^18^ In GC, this convergence is experimentally visible as G3BP1–YWHAZ-mediated Bax retention.^33^ For BAD, Bim, Mcl-1 and broader BCL-2 family interactions, digestive-cancer-specific validation is less mature; these mechanisms are best framed as background biology unless a direct digestive-cancer module is demonstrated.

The second convergence point is compartmentalization. SG biology depends on cytoplasmic condensates; UPR depends on transcriptional and translational remodeling; autophagy depends on lysosomal and cytoplasmic membrane trafficking; anoikis resistance depends on receptor localization and mitochondrial signaling. Examples include Bax cytoplasmic retention in GC, YY1 cytoplasmic retention in CRC, TFEB cytoplasmic sequestration in TGF-β/SMAD4-driven CRC epithelial contexts, and EGFR stabilization during detachment stress in HCC.^25-27^ ^,^ ^33^ These examples justify localization-aware assays as core readouts.

The third convergence point is residual disease. Cells that survive an initial stress episode may not return fully to their pre-stress state. In this context, stress memory refers to a persistent post-stress state in which surviving cells retain altered signaling, localization or transcriptional programs after the initial stress has subsided, potentially enhancing survival during subsequent stress exposure or residual-disease progression. SG components, UPR genes, autophagy machinery, receptor-trafficking programs and YAP1–RRM1/RRM2 signaling may remain primed or become enriched in residual clones. Direct evidence for durable 14-3-3-mediated stress memory in digestive cancers remains limited; nevertheless, the existing modules support the hypothesis that post-treatment residual disease may be enriched for specific 14-3-3–client states, including G3BP1–YWHAZ–Bax, SFN–YY1–UPR and SFN–YAP1–RRM1/RRM2.^25^ ^,^ ^33^ ^,^ ^55^ Paired pre- and post-treatment specimens provide the most direct approach for testing this hypothesis.

## 5. Biomarker framework and patient stratification

A clinically useful 14-3-3 biomarker is best defined as a stress-state biomarker rather than a simple expression marker. High YWHAZ expression does not necessarily indicate active G3BP1–YWHAZ–Bax sequestration. High SFN expression does not necessarily indicate UPR dependence unless YY1 is cytoplasmic and UPR genes are active. High EGFR expression does not necessarily indicate SFN–EGFR/ERK-dependent anoikis resistance unless detachment stress stabilizes receptor signaling.^25^ ^,^ ^27^ ^,^ ^33^

A practical biomarker framework includes three layers. The first layer is expression of the relevant 14-3-3 isoform or partner protein, such as YWHAZ/14-3-3ζ, SFN/14-3-3σ, G3BP1, YAP1, YWHAH/14-3-3η, EGFR or RRM1/RRM2. The second layer is localization or complex formation, such as Bax cytoplasmic retention, YY1 cytoplasmic retention, TFEB sequestration, SFN-YAP1 interaction, EGFR stabilization or YWHAH-linked MAPK/ERK signaling. The third layer is the stress-output phenotype, such as SG abundance, UPR gene signature, autophagy flux, suspension survival, RRM1/RRM2 expression, caspase activation or chemotherapy response. A module is most convincingly active when these layers align. A practical validation framework for stress-state 14-3-3 biomarkers is outlined in Table 2.

**Table 2.** Stress-state biomarker and validation framework.

Validation layer	Representative readouts	Purpose
Expression	YWHAZ/14-3-3ζ, SFN/14-3-3σ, G3BP1, YWHAH/14-3-3η, YAP1, EGFR, RRM1/RRM2	Defines whether the candidate module can exist in a given tumor.
Complex or localization	Bax cytoplasmic retention; YY1 cytoplasmic retention; TFEB sequestration; SFN–YAP1 interaction; EGFR stabilization; G3BP1–YWHAZ proximity	Determines whether the 14-3-3–client module is active rather than merely expressed.
Stress-output phenotype	SG abundance; UPR signature; autophagy flux; suspension survival; RRM1/RRM2 induction; caspase activation; chemotherapy response	Links the module to functional resistance or survival.
Stress-matched model	Drug pulse; ER stress; nutrient deprivation; hypoxia; suspension culture; spheroids; organoids; stromal/EV co-culture	Tests the module under the condition in which it is expected to matter.
Clinical validation	paired pre- and post-treatment specimens; residual disease sampling; spatial profiling; EV-tissue correlation; outcome association	Moves the module from mechanistic observation to patient stratification.

Sample selection is central. Pretreatment biopsies may identify baseline module potential, but post-treatment samples may reveal resistance programs more directly. GC tumors after platinum exposure may show stronger SG-associated biology than pretreatment tissue. CRC residual lesions after chemotherapy may be enriched for SFN–YY1–UPR signaling. PDAC after gemcitabine exposure may show increased SFN–YAP1–RRM1/RRM2 activity. HCC or CCA invasive fronts may better reveal anoikis modules than bulk tumor cores.

EV-based biomarkers are attractive because digestive cancer tissue can be difficult to sample repeatedly. BMSC-derived exosomes have been linked to GC oxaliplatin resistance through G3BP1–YWHAZ-associated signaling, and serum extracellular vesicle stratifin has been reported as a biomarker of perineural invasion in stage II CRC.^34^ ^,^ ^68^ However, EV biomarkers require rigorous standardization of isolation method, cargo normalization and correlation with tissue localization. EV signals are best integrated with tissue and functional evidence rather than treated as standalone surrogates.

## 6. Translational implications and validation pipeline for interaction-directed therapy

The therapeutic implication of this framework is not global inhibition of all 14-3-3 proteins. Pan-family inhibition could disrupt both pro-survival and tumor-suppressive modules and may impair normal stress responses.^13^ ^,^ ^16^ Recent high-throughput screening work suggests that small molecules capable of disrupting selected 14-3-3 protein interactions can be identified.^69^ The rational therapeutic unit is a stress-state-specific 14-3-3–client interaction. Candidate examples include G3BP1–YWHAZ–Bax in GC, SFN–YY1–UPR in CRC, SFN–YAP1–RRM1/RRM2 in PDAC and SFN–EGFR/ERK in HCC.^25^ ^,^ ^27^ ^,^ ^33^ ^,^ ^55^

In GC, the most direct strategy is disruption of SG-associated apoptotic sequestration. The core rationale is the direct G3BP1–YWHAZ–Bax chemoresistance mechanism.^33^ BMSC-derived exosome data provide a supportive model for microenvironmental amplification of this pathway.^34^ Preclinical testing can determine whether chemotherapy combined with SG disruption, G3BP1–YWHAZ interference or apoptosis priming restores Bax mitochondrial access and cell killing. Useful validation systems include GC organoids, platinum or fluoropyrimidine pulse exposure, G3BP1/YWHAZ proximity assays, Bax localization, caspase activation and clinical correlation with adjuvant chemotherapy outcome.

In CRC, therapy development needs to distinguish the SFN–YY1–UPR survival module from tumor-suppressive SFN modules. UPR modulation is most rationally tested in SFN-high/YY1-cytoplasmic/UPR-high models rather than in unselected CRC systems.^25^ General UPR-therapy and CRC-UPR literature can guide pathway selection, but it cannot replace direct SFN–YY1 evidence.^39^ ^,^ ^41^ Conversely, SFN–TFEB and SFN–NEDD4L–HIF-1α observations warn against simple SFN inhibition in tumors where SFN restrains autophagy-driven plasticity or hypoxia-induced metastasis.^26^ ^,^ ^46^

In PDAC, gemcitabine-based therapy could be combined with strategies that target replication-stress tolerance, ribonucleotide reductase, YAP/TAZ signaling or apoptosis priming in SFN–YAP1–RRM1/RRM2-high models. The direct 14-3-3 evidence comes from SFN-YAP1 interaction and acquired gemcitabine-resistance data.^54^ ^,^ ^55^ The broader rationale for RRM1/RRM2 and YAP/TAZ targeting is supported by separate literature.^51^ ^,^ ^53^ ^,^ ^56^ ^,^ ^59^ Existing literature on BCL-2 family proteins and BH3 mimetics provides a general rationale for lowering the apoptotic threshold,^67^ ^,^ ^70^ ^,^ ^71^ but pancreatic-specific combinations require validation in drug-exposed organoids and stromal co-cultures.^72-74^

In HCC and CCA, therapeutic testing is most informative when focused on detachment survival. HCC models with SFN-dependent EGFR stabilization may be suitable for EGFR/ERK pathway modulation under suspension or metastatic-stress conditions.^27^ In CCA, SFN-associated anoikis data require client mapping and stress-state assays before pathway inhibition can be proposed.^63-65^

A robust preclinical pipeline includes four steps. First, module activation is confirmed using localization, proximity or interaction assays. Second, the candidate client interaction is perturbed genetically or pharmacologically. Third, the perturbation is tested under the relevant stress condition, such as chemotherapy pulse exposure, ER stress, nutrient deprivation, hypoxia, suspension culture, spheroids, organoids or stromal co-culture. Fourth, the module is validated in patient-derived models and clinical specimens. This sequence is more informative than screening generic stress-pathway inhibitors in unselected models.

## 7. Methodological considerations and future directions

Precise isoform nomenclature is essential. YWHAZ/14-3-3ζ, SFN/14-3-3σ, YWHAH/14-3-3η and other isoforms are not interchangeable. Conclusions from one isoform cannot be generalized to the whole family without direct evidence. This is particularly important in CRC, where SFN can support YY1-UPR-mediated therapy tolerance,^25^ participate in p53-dependent checkpoint control,^42-45^ restrain TFEB-associated autophagy^26^ or promote HIF-1α degradation through NEDD4L.^46^ SFN nomenclature requires special care. In cancer biology, SFN usually refers to stratifin/14-3-3σ, whereas in nutritional and chemoprevention literature SFN often refers to sulforaphane.

A second limitation is that total 14-3-3 abundance does not necessarily indicate functional dependence. Because 14-3-3 activity depends on client phosphorylation, complex formation and localization, phosphosite mapping and 14-3-3–client binding assays are central mechanistic readouts.^14^ ^,^ ^23^ ^,^ ^75^ Additional approaches, including co-immunoprecipitation, proximity ligation, proximity labeling, protein-fragment complementation and spatial proteomics, can be used to define active complexes in stress-matched models. These assays are most informative when performed under stress-matched conditions: SG-mediated chemoresistance requires drug exposure and SG markers; UPR-mediated tolerance requires proteotoxic or ER stress conditions; and anoikis resistance requires detachment or suspension models.

Translational studies will benefit from combining paired specimens, single-cell and spatial methods, EV profiling and patient-derived organoids. Spatial and single-cell methods can help map where SG, UPR, autophagy and anoikis-related modules operate, while organoids and co-culture systems can test causality.^72-74^ ^,^ ^76-79^ The most immediate clinical applications are likely to be biomarker-guided combinations rather than 14-3-3 monotherapy. Peptides, small molecules, molecular glues or degraders that selectively modulate disease-relevant 14-3-3–client interactions may eventually provide better specificity than pan-family inhibition.^69^

## 8. Conclusions

14-3-3 proteins are best understood in digestive cancers as stress-adaptive client-specific adapters rather than as uniformly oncogenic or tumor-suppressive factors. The strongest current evidence supports four interaction-centered modules: G3BP1–YWHAZ–Bax in GC chemoresistance, SFN–YY1–UPR in CRC therapy tolerance, SFN–YAP1–RRM1/RRM2 in PDAC gemcitabine resistance and SFN–EGFR/ERK in HCC anoikis resistance. Additional CRC modules, including SFN–TFEB, SFN–NEDD4L–HIF-1α, YWHAH–MAPK/ERK-autophagy and SFN-associated anoikis in CCA, highlight context dependence and require explicit evidence grading.

The main translational message is that 14-3-3 expression is not sufficient as a biomarker or target. A clinically actionable module is best defined by isoform or partner expression, client localization or complex formation, and stress-output function. Such a framework may guide biomarker-enriched preclinical testing of interaction-specific combinations and help avoid the biological risks of indiscriminate 14-3-3 inhibition.

## Data Availability

No new datasets were generated or analyzed in this narrative review.

## Abbreviations

AKT: protein kinase B
BAD: BCL-2-associated agonist of cell death
Bax: BCL-2-associated X protein
BCL-2: B-cell lymphoma 2
BMSC: bone marrow mesenchymal stromal cell
CCA: cholangiocarcinoma
CRC: colorectal cancer
EGFR: epidermal growth factor receptor
ER: endoplasmic reticulum
ERK: extracellular signal-regulated kinase
EV: extracellular vesicle
GC: gastric cancer
G3BP1: G3BP stress granule assembly factor 1
HCC: hepatocellular carcinoma
HIF-1α: hypoxia-inducible factor 1 alpha
MAPK: mitogen-activated protein kinase
mTOR: mechanistic target of rapamycin
NEDD4L: NEDD4-like E3 ubiquitin protein ligase
PDAC: pancreatic ductal adenocarcinoma
PI3K: phosphoinositide 3-kinase
RRM1: ribonucleotide reductase catalytic subunit M1
RRM2: ribonucleotide reductase regulatory subunit M2
SG: stress granule
TAZ: transcriptional coactivator with PDZ-binding motif (WWTR1)
TFEB: transcription factor EB
UPR: unfolded protein response
YAP1: Yes-associated protein 1
YY1: Yin Yang 1
